# Placental fibroblast growth factor 21 is not altered in late-onset preeclampsia

**DOI:** 10.1186/s12958-015-0006-3

**Published:** 2015-03-08

**Authors:** Marloes Dekker Nitert, Katherin Scholz-Romero, Marta H Kubala, H David McIntyre, Leonie K Callaway, Helen L Barrett

**Affiliations:** School of Medicine, The University of Queensland, Butterfield Street, Herston, 4029 QLD Australia; The University of Queensland Centre for Clinical Research, The University of Queensland, Herston, QLD Australia; Obstetric Medicine, Royal Brisbane and Women’s Hospital, Herston, QLD Australia; Mater Health Services, South Brisbane, QLD Australia

**Keywords:** FGF21, Preeclampsia, Placenta, PPAR-alpha, PPAR-gamma, GLUT

## Abstract

**Background:**

Preeclampsia (PE) is associated with alterations of placental function. The incidence of PE is higher in insulin resistant states. Women with PE have high circulating levels of the metabolic regulator fibroblast growth factor 21 (FGF21). FGF21 is synthesized in the placenta. The aim of this study was to compare the expression of FGF21, its receptors, downstream targets and transcriptional regulators in placental tissue from pregnancies with and without late-onset PE. Circulating FGF21 in maternal and cord blood was also studied.

**Methods:**

mRNA expression was determined by semi-quantitative real-time PCR and normalized for cellular composition in 17 women with and 20 without PE. Protein expression was quantified by Western Blot. FGF21 levels were measured by ELISA in maternal and cord serum of ten mother-baby dyads per condition.

**Results:**

Placental FGF21 mRNA and protein expression were similar in PE compared with control. Placental mRNA expression of the FGF receptors (1–4) and the co-receptor beta-Klotho was not different between the groups. There was no difference in the expression of the glucose transporters GLUT1, 3 or 4. PPAR-alpha but not PPAR-gamma expression was decreased in PE. Maternal FGF21 serum levels were not significantly different in PE. FGF21 was detected in cord blood of 6 infants (4 PE, 2 controls) but was undetectable in 14 infants.

**Conclusions:**

Late-onset PE is not associated with major changes to the expression of FGF21, its receptors or metabolic targets.

**Electronic supplementary material:**

The online version of this article (doi:10.1186/s12958-015-0006-3) contains supplementary material, which is available to authorized users.

## Background

Preeclampsia (PE), a syndrome of hypertension and proteinuria, is a common complication of pregnancy affecting 2 to 8% of pregnancies worldwide [1]. The diagnostic criteria include hypertension with blood pressures > 140/90 mmHg and proteinuria developing after 20 weeks gestation. PE (in particular early-onset PE diagnosed prior to 34 weeks gestation) is associated with adverse outcomes for mother and baby including preterm delivery, stillbirth and intrauterine growth restriction. In the long term, women with PE complicating their pregnancy have higher risks of developing hypertension, cardiovascular disease and type 2 diabetes [2,3].

The risk of developing PE is higher in women with increased insulin resistance, as occurs in obesity and gestational diabetes mellitus [4]. Recently, there has been significant interest in a newly discovered metabolic regulator, fibroblast growth factor 21 (FGF21). FGF21 increases insulin sensitivity, glucose and lipid metabolism [5-8]. FGF21 is synthesized and secreted from the liver, adipose tissue, pancreatic β-cells, skeletal muscle, white blood cells and possibly cerebrospinal fluid [9-12]. Recent reports have shown that circulating levels of FGF21 at 28 weeks gestation are increased in women with PE compared with controls [13]. This suggests that FGF21 might have a role in the regulation of metabolism, and provide some explanation of the link between insulin resistance and PE.

We have previously shown that FGF21 is synthesized in the placenta [14]. The placenta not only transfers nutrients but also actively synthesizes and secretes a large number of molecules, such as FGF21. It could be useful to understand the role of these molecules in pregnancy pathology. FGF21 can bind to all four isoforms of the FGF-receptor (FGFR), although preferentially to FGFR1. The co-receptor β-klotho increases binding affinity between FGF21 and its receptor [15]. Human placenta expresses all four FGF receptor isoforms in addition to β-klotho [14]. In placenta, FGF21 mRNA expression is positively correlated with increased expression of glucose transporters (GLUT) [14]. Transcriptional regulation of FGF21 is under the control of peroxisome proliferator-activated receptors (PPARs) α and γ [16,17]. In the placentae of women with an uncomplicated pregnancy or gestational diabetes mellitus, there was a positive correlation between the gene expression levels of PPARα and FGF21 and a tendency for a positive correlation between PPARγ and FGF21 [14].

Since the placenta synthesizes FGF21, it has been hypothesized that it could be involved in the regulation of placental metabolism, which may affect infant growth and development through differential expression of nutrient receptors and transporters. Given the relationship between insulin resistance and PE, the role FGF21 plays may be critical. To date, it is not clear whether placental FGF21 is differentially expressed in preeclampsia and if this could contribute to altered placental metabolism.

This study investigated whether expression of placental FGF21, its receptors and co-receptor as well as its transcriptional regulators and metabolic targets differed in preeclampsia as compared to normal pregnancy. Circulating FGF21 in maternal blood and infant cord serum was also measured in mother-baby dyads to assess if PE affects these levels.

## Methods

### Participants

Pregnant women were enrolled with informed consent in the third trimester of pregnancy, with approval from the human research ethics committees from the Royal Brisbane and Women’s Hospital and The University of Queensland. Seventeen women were diagnosed with preeclampsia according to the SOMANZ research criteria from 2008, consisting of two blood pressure measurements of >140/90 separated by >2 hours and the presence of proteinuria [18]. A control group of 20 normotensive women was selected with matching for pre-pregnancy BMI, gestational age at delivery, baby gender, birth weight and maternal age. Women with diabetes were excluded from this study. Placental samples and cord blood samples were collected at delivery. One cm^3^ samples were taken from the maternal and fetal sides of the placenta in areas away from calcifications and visible anatomical abnormalities, snap-frozen in liquid nitrogen and stored at −80°C. Cord blood was spun for 10 minutes at room temperature at 3000xg, serum was collected and stored at −80°C until use. For immunohistochemistry, 1 cm^3^ samples were obtained from both sides of the placenta, washed in PBS, preserved in 4% paraformaldehyde for 72 hours until transfer to saturated sucrose until paraffin embedding.

### Gene expression analysis

Total RNA was isolated from the frozen placental samples with the DNA/RNA AllPrep kit (Qiagen) according to the manufacturer’s instructions. The samples were lysed in lysis buffer through agitation with a 5 mm stainless steel ball for 2x2 minutes at 30 Hz in the TissueLyser II (Qiagen). 750 ng of total RNA 260/280 ratios > 1.8 was transcribed to cDNA with the QuantiTect Reverse Transcription kit (Qiagen) employing a mixture of random hexamers and oligodT primers according to the manufacturer’s recommendations.

Primers were designed for each target gene with the NCBI primer design tool, ensuring coverage of an exon-exon junction in one of the primers of the primer pair. All primer sequences and products were blasted and were specific for the target gene only. The primer sequences and amplicon lengths are listed in Additional file 1: Table S1.

18.75 ng of cDNA, 300 nM of forward and reverse primer and 10 μl iTaq universal SYBR green PCR master mix (BioRad) was used per QPCR reaction in a iQ5 QPCR machine (BioRad). Cycling conditions consisted on 1 cycle at 95°C for 10 min, 40 cycles of 15 seconds at 95°C and 1 minute at 59°C, followed by melt curve analysis.

Target gene expression was normalized to the geometric mean of the expression of the endogenous control gene *TBP*, and the expression of the cellular markers *CK7* (trophoblasts), *CD34* (endothelial cells), and *DES* (smooth muscle cells) to adjust for potential differences in cellular composition of the placental samples. Target gene expression was analyzed with the ΔΔCt method after ensuring amplication efficiency varied between 90 and 110%.

### Protein expression analysis

Protein was isolated from placental samples by agitation with a 5 mm stainless steel ball in RIPA buffer for 2×2 minutes at 30 Hz in the TissueLyserII. Supernatant was obtained, spun for 10 min at 4°C at 12000 rpm to remove cellular debris. Protein content was measured with the BCA method.

Thirty μg of protein was loaded onto a 4-12% NuPAGE® BIs-Tris gel (Life Technologies, Mulgrave, VIC, Australia), run and transferred to a polyvinylidene difluoride membrane (Millipore, Kilsythe, VIC, Australia). Non-specific binding of antibodies was blocked by preincubating the membranes for 60 minutes in 5% non-fat dry milk dissolved in TBS-Tween. The blots were incubated overnight at 4°C with the primary antibodies, washed and incubated for 1 hour at room temperature with the secondary antibodies. The LI-COR system was used enabling simultaneous visualization of the target protein and the loading control (β-actin). Densitometric analysis was performed with the Odyssey Infrared Imaging system.

Primary polyclonal rabbit anti-human FGF21 (ab64857, Abcam, Cambridge MA, USA) was diluted to 1 μg/ml and mouse monoclonal anti-human β-actin (A5316, Sigma Aldrich, Castle Hill, NSW Australia) was diluted 1:20000 in 5% non-fat dry milk/PBS-Tween. Secondary LiCOR antibodies were diluted at 1:10000 for goat anti-rabbit 800CW (926–32211, LI-COR) and at 1:15000 for donkey anti-mouse 680LT (926–68022, LI-COR).

### Protein localization

Immunohistochemistry was used to localize FGF21 expression to the placenta. Five μm thick sections of paraffin-embedded placental samples (N = 4 for PE and controls each) were baked and dehydrated. Antigen retrieval was performed by incubation of the samples in citrate buffer (10 mM, pH6.0) for 30 minutes. Endogenous hydrogen peroxidase activity was blocked by incubating the slides for 10 min with 3% hydrogen peroxide followed by 15 min with the universal non-specific background-blocking agent Biocare Background Sniper (MACH2, Biocare Medical, Concord CA, USA). The slides were incubated overnight with primary antibody, human rabbit anti-human FGF21 (ab54857, Abcam) at 1:300 at 4°C. The slides were washed and incubated with rabbit HRP-polymer (MACH2, Biocare Medical) for 60 minutes at room temperature. HRP activity was visualized by DAB and the slides were counterstained with Harris’ Haematoxylin (HHS16, Sigma Aldrich) and mounted with coverslips. To ensure antibody specificity, staining was performed in the absence of primary antibody or in the presence of IgG control as primary antibody yielding similar patterns of background staining.

### Circulating FGF21 levels

Maternal and cord blood serum samples of 10 mother-baby dyads were analyzed in duplicate for human FGF21 by ELISA (ab125966, Abcam) following the manufacturer’s recommendations. The detection limit of this assay is 0.03 ng/mL, the intra-assay variability is 4.8% and the inter-assay variability is 7.4%.

### Statistical analysis

The participant characteristics, which are normally distributed as tested by the D’Agostino & Pearson omnibus normality test, are reported as means ± SD and compared with Student’s *t*-tests. The experimental data were tested for adherence to the normal distribution and did not conform. These data were therefore reported as median (interquartile range IQR) and all statistical analyses were performed with non-parametric methods: A Kruskal-Wallis one-way analysis of variance by ranks test for the comparisons between groups and Spearman’s rank correlation tests for correlations. Dunn’s multiple comparisons test was applied to correct for multiple testing where necessary.

## Results

### Study population

Seventeen women with and 20 women without PE were recruited for this study. The women were matched for gestational age of delivery, maternal age, pre-pregnancy BMI and gender of the baby (Table 1). The women with PE had significantly higher systolic and diastolic blood pressure at enrolment in the study, even with 11 women on treatment with anti-hypertensive agents. The women with PE also had a tendency for higher systolic blood pressure in early pregnancy (115 +/− 14 vs. 107 +/− 8 mmHg, *P* = 0.08 for normotensive women). Maternal blood glucose values in response to a 50 g glucose challenge test were similar in both groups and none of the women were diagnosed with gestational diabetes mellitus.

**Table 1:** **Clinical characteristics of the study participants**

	**Control**	**PE**	**P-value**
N	20	17	
Age (years)	32.8 ± 5.3	30.2 ± 6.7	0.25
Pre-pregnancy BMI (kg/m2)	24.9 ± 4.9	25.2 ± 5.5	0.87
Gravidity (median, IQR)	2.5 (2–4)	2 (1–4)	ND
Parity (median, IQR)	1 (1–2)	0 (0–1)	ND
Ethniticy (Caucasian/other)	18/2	17/0	ND
SBP (T3) mmHg	118 ± 13	135 ± 18*	0.003
DBP (T3) mmHg	73 ± 9	84 ± 15*	0.01
SBP (early preg) mmHg	107 ± 8	115 ± 14	0.08
DBP (early preg) mmHg	65 ± 8	67 ± 10	0.40
Mode of delivery (VD/CS)	0/20	10/7	<0.0001
GA delivery (d)	269.6 ± 8.3	268.1 ± 10.3	0.64
Birth weight (g)	3279 ± 623	3150 ± 673	0.55
Baby gender (M/F)	10/10	9/8	ND
Glucose screen result (mmol/L)	5.76 ± 1.02	6.10 ± 1.46	0.51

Results expressed as mean ± SD unless indicated otherwise; ND, not determined; VD, vaginal delivery; CS, Caesarean section; *11 of the 17 treated with anti-hypertensive agents.

### FGF21 expression in preeclampsia

**Figure 1:**
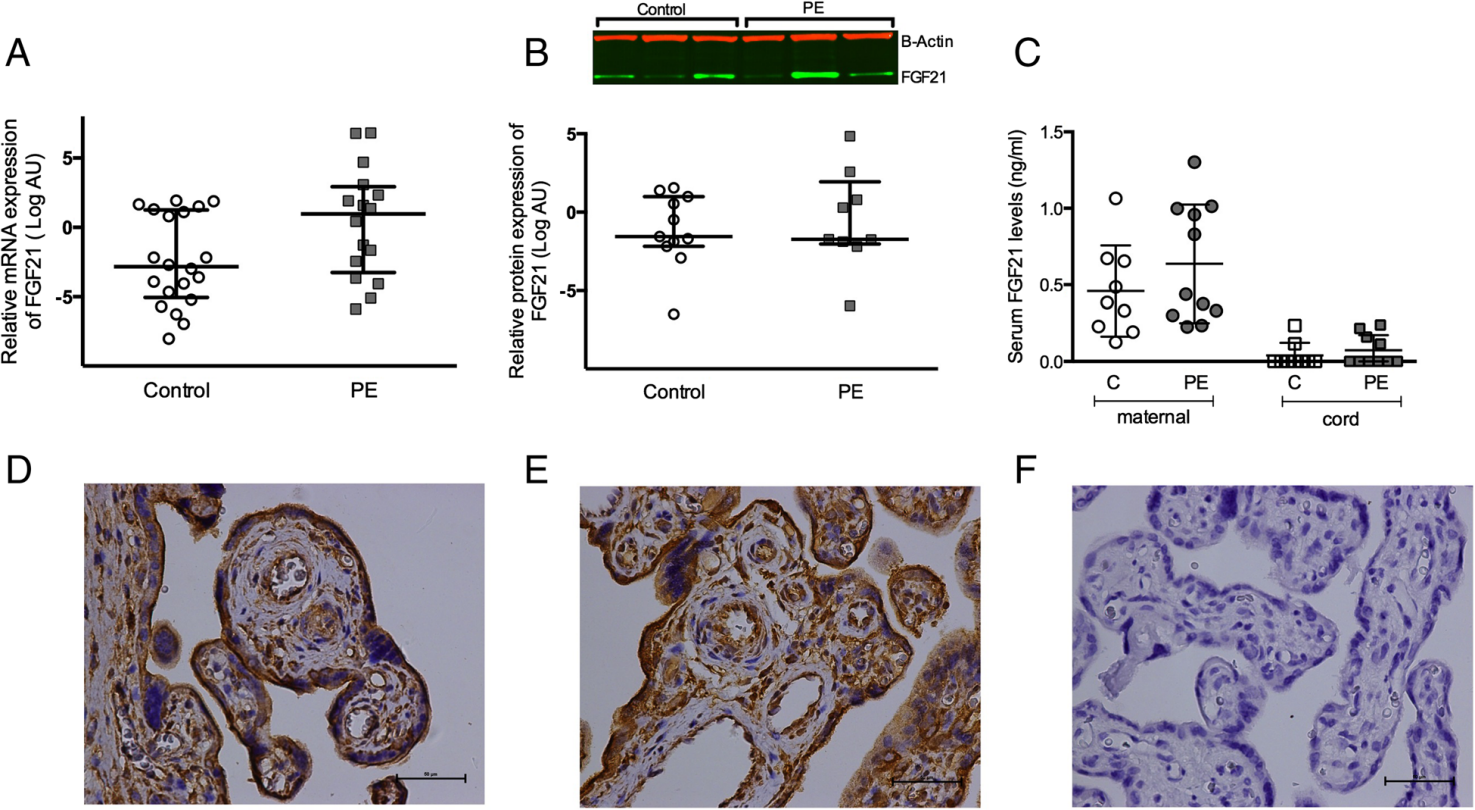
**FGF21 in PE. A)** Placental mRNA expression of FGF21 in 20 control women (white circles) and 17 women with PE (grey squares). **B)** Placental protein expression of FGF21 in 11 control women (white boxes) and 9 women with PE (grey boxes), inset representative picture of western blot with FGF21 in green and B-actin in red. **C)** Serum levels of FGF21 of in maternal and cord blood of 10 control women (white circles and white squares respectively) and 10 with PE (grey circles and grey squares respectively). Representative immunohistochemistry of FGF21 in placenta from control women **(D)**, women with PE **(E)** and no primary antibody negative control **(F)**. Line is median and IQR in **A** and **B**; *, P < 0.05.

Placental FGF21 mRNA expression was not significantly increased in women with PE (PE median 1.96 (IQR 0.11-7.60) vs. controls 0.14 (0.03-2.37), *P* = 0.24) (Figure 1A). Protein levels for FGF21 were not significantly altered in PE (PE 0.30 (0.25-3.85) vs. controls 0.34 (0.22-1.99), *P* = 0.87) (Figure 1B). The inter-individual variation in gene and protein expression of FGF21 was extensive but was not correlated to maternal factors such as pre-pregnancy BMI, gestational age at delivery or birth weight. FGF21 protein was localized to trophoblasts, endothelial cells, stromal cells (Figure 1D and E) and decidual cells (Additional file 2: Figure S1). There was no apparent difference in the protein localization or amount between placental tissue from women with or without PE.

Circulating FGF21 was examined in maternal and cord blood samples of 10 mother-baby dyads in each group. FGF21 levels in maternal blood were similar in women with and without PE (PE 0.44 (0.30-1.00) ng/ml vs. control 0.38 (0.21-0.66), *P* = 0.38) (Figure 1C). Maternal FGF21 levels were correlated to maternal systolic blood pressure at enrolment (rho 0.50, *P* = 0.02) but not to diastolic blood pressure (rho 0.39, *P* = 0.09) or prepregnancy BMI (rho −0.04, *P* = 0.87). Cord blood FGF21 fell below the level of detection (0.03 ng/ml) in most cord blood samples but was just above the level of detection in 2 cord blood samples of normotensive pregnancies and 4 cord blood samples of PE pregnancies. Cord blood FGF21 levels were not correlated to maternal FGF21 levels.

### FGF receptors

**Figure 2:**
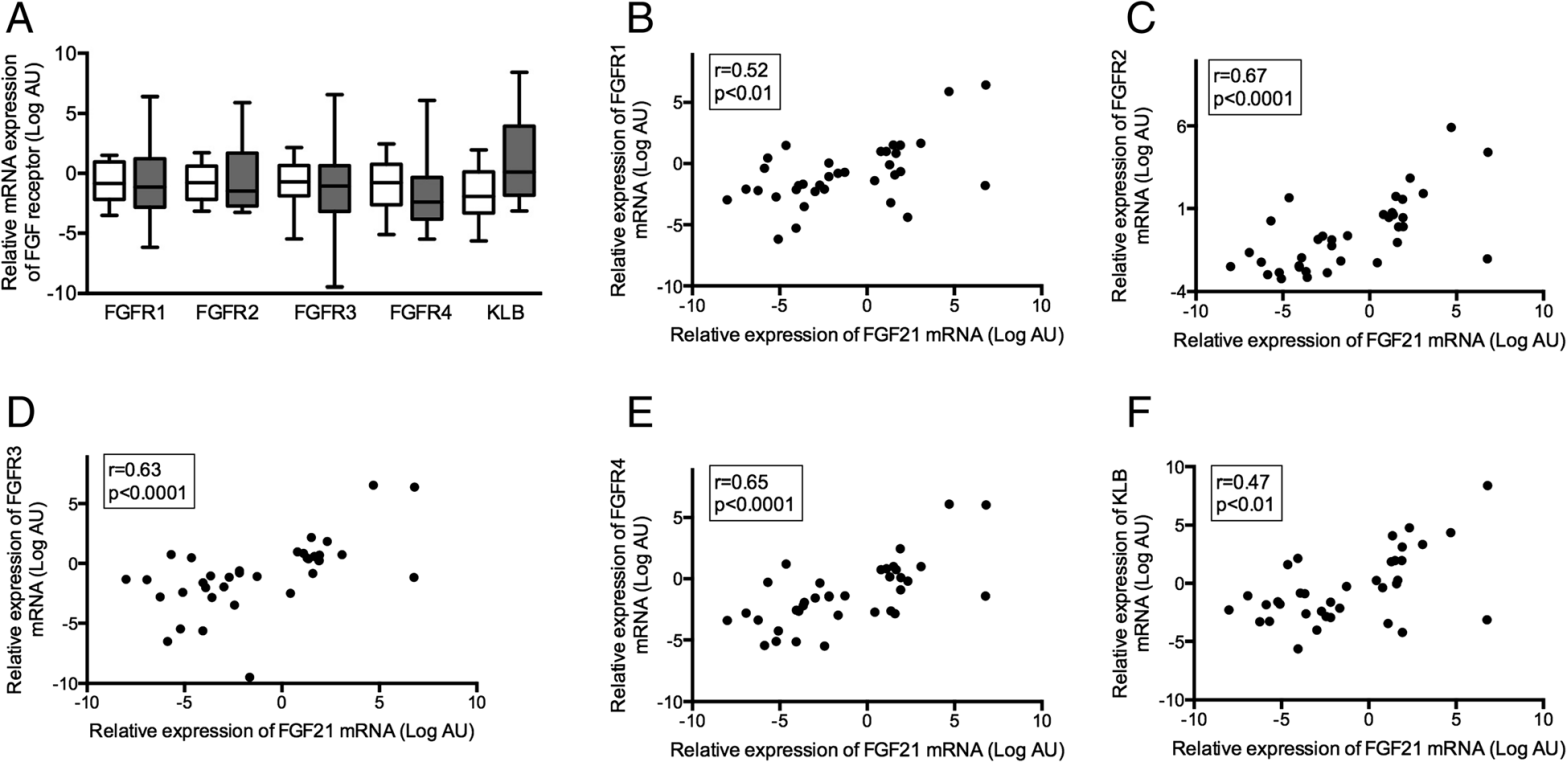
**Placental FGF receptors in PE. A)** Placental mRNA expression of FGF receptor isoforms 1–4 and the co-factor β-klotho (KLB) in 20 control women (white boxes) and 17 women with PE (grey boxes). Boxes, median (IQR); whiskers 2.5-97.5% CI; *, P < 0.05. Correlations between placental mRNA expression of FGF21 and FGFR1 **(B)**, FGFR2 **(C)**, FGFR3 **(D)**, FGFR4 **(E)** and KLB **(F)** including 20 women with and 17 women without PE.

FGF21 can bind to all four isoforms of the FGF receptor family with a preference for FGFR4 especially in the presence of the co-receptor β-klotho (*KLB*). In PE, placental expression of each of the FGFR isoforms was not different from control pregnancies (Figure 2A): FGFR1 mRNA expression in PE 0.45 (0.14-2.33) vs. control 0.56 (0.15-1.30), *P* = 0.99; FGFR2 PE 0.36 (0.15-3.21) vs. control 0.59 (0.22-1.52), *P* = 0.99; FGFR3 PE 0.48 (0.11-1.56) vs. control 0.61 (0.27-1.59), *P* = 0.99; and FGFR4 PE 0.19 (0.07-0.79) vs. control 0.58 (0.16-1.68), *P* = 0.99. Placental mRNA expression for the co-factor β-klotho was not significantly increased in PE (1.07 (0.28-15.31)) vs. control 0.26 (0.10-1.09), *P* = 0.13. However, FGF21 mRNA expression correlated significantly and positively with the expression of all FGFR isoforms (Figure 2B-E) as well as β-klotho (Figure 2F).

### PPAR mRNA expression in preeclampsia

**Figure 3:**
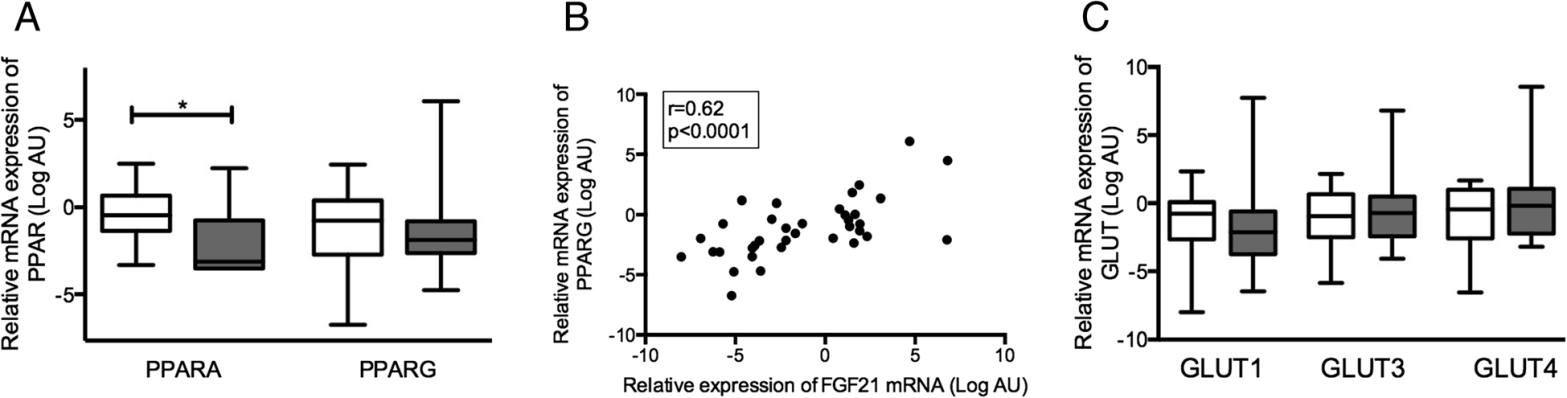
**PPAR and GLUT expression in placenta in PE. A)** Placental mRNA expression of PPARA and PPARG in 20 control women (white boxes) and 17 women with PE (grey boxes). **B)** Correlation between placental mRNA expression of FGF21 and PPARG. **C)** Placental mRNA expression of the glucose transporter isoforms GLUT1, 3 and 4. Boxes, median (IQR); whiskers 2.5-97.5% CI; ***, P < 0.001.

FGF21 mRNA expression is regulated by PPARα and γ in different tissues. Placental mRNA expression of PPARα and γ was investigated in our cohort of PE women. PPARα expression in the placenta was significantly decreased in PE (0.12 (0.09-0.59)) compared with controls (0.73 (0.39-1.57)), *P* = 0.049. Placental PPARγ mRNA expression was not different between women with (0.27 (0.16-0.57)) or without PE (0.59 (0.15-1.30)), *P* = 0.99 (Figure 3A). FGF21 mRNA expression was however positively correlated to PPARγ mRNA expression (Spearman’s ρ = 0.62, *P* < 0.0001) (Figure 3B) but not to PPARα mRNA expression (Spearman’s ρ = −0.09, *P* = 0.62).

### Glucose transporter mRNA expression in preeclampsia

Glucose transporters are targets of FGF21 signaling. We analyzed the gene expression of the glucose transporters GLUT1, GLUT3 and GLUT4 in the placentas from women with and without PE. There were no significant differences in the expression of the GLUTs between the groups (Figure 3C). The expression levels of FGF21 mRNA were positively correlated with those of GLUT1 (Spearman’s ρ = 0.50, *P* = 0.0019), GLUT3 (Spearman’s *P* = 0.74, *P* < 0.0001) and GLUT4 (Spearman’s ρ = 0.58, *P* = 0.0002).

## Discussion

The aim of the current study was to investigate whether FGF21, its receptors and transcriptional activators and downstream targets in the placenta were altered in the setting of PE. No changes were seen in placental FGF21 mRNA levels, its protein level, localization and expression of its receptors, co-receptor or downstream targets GLUT1, 3, and 4 was not changed. PE was however associated with decreased placental expression of PPARα and unaltered PPARγ expression. The serum levels of FGF21 were not different in PE.

### Placental FGF21, receptors and cofactors

Placental FGF21 mRNA and protein expression is highly variable in normal pregnancies as well as in pregnancies affected by late-onset preeclampsia (current study) or gestational diabetes mellitus [14]. In the current study we found no changes in mRNA and protein levels, or localization. The positive correlations between the placental expression of FGF21 and its receptors and co-factor found in the current study could suggest that they may be regulated by common mechanisms and that interindividual variations in the expression of members of the FGF21 pathway in the placenta are common. This may result in small differences in lipid and glucose metabolism in the placenta between women. This could occur despite unchanged expression of glucose transporters: it has been argued that placental glucose transport is not limited by transport capacity but rather by blood flow [19].

### Placental PPARs

In the current study, PPARα mRNA levels were significantly decreased in PE. In contrast, we recently reported that in placentae from women with GDM, there is an increase in PPARα [14]. PPARγ was not significantly decreased in the placentae of women with late-onset PE. Previous studies have variably reported an increase in PPARα and decrease of PPARγ expression in PE [20] or no change [21,22]. These differences may be due to differences in disease severity, gestational age, BMI and ethnicity. PE combined with intrauterine growth restriction, which suggests more severe pathology, is associated with larger differences in PPAR expression [21]. In contrast to our previous results in placentae from women with or without gestational diabetes [14], PPARα expression is not correlated to FGF21 expression. This could indicate that the regulation of FGF21 mRNA expression is altered in PE.

### GLUT in placenta

The placenta expresses the glucose transporters GLUT1, 3 and 4 [23-25]. GLUT expression in PE has not been studied in detail previously. Placental expression of GLUT3 is increased in idiopathic intrauterine growth restriction [26]. In gestational diabetes mellitus, an increase in the placental expression of GLUT3 and 4 has been observed [14]. GLUT3 in intrauterine growth restriction was colocalized with the hypoxic transcription factor HIF-1α [26] whereas FGF21 has been reported to induce GLUT1 expression in adipocytes [27]. HIF-1α expression is increased in preeclampsia [28,26]. It may therefore be that the regulation of glucose transporters is regulated differently in gestational diabetes mellitus and the hypoxic conditions of PE and intrauterine growth restriction.

### Serum FGF21

Serum FGF21 levels are highly variable both in pregnancy [13] and outside of pregnancy [29-31]. Maternal serum FGF21 levels have previously been reported to be increased in PE [13]. In our study, we did not observe a significant change in maternal FGF21 levels. Overall, the serum concentration of FGF21 in our samples was similar to recent studies [13,32,10]. The lack of an elevation in FGF21 in the maternal serum of women with PE in our study may be due to differences in the characteristics of the study participants such as BMI, insulin resistance, gestation at blood sampling and parity [31]. Most of the women included in the current study were not obese, and FGF21 serum levels did not correlate with pre-pregnancy BMI. The higher glucose screen results indicate the higher level of insulin resistance in the women who went on to develop PE. This is consistent with previous studies showing a positive association between PE and insulin resistance (reviewed in [33]). Circulating FGF21 in pregnancy has previously been positively correlated with fasting insulin, triglycerides and leptin levels but negatively with LDL cholesterol in univariate analyses [13]. In multivariate analyses, the associations with LDL cholesterol and triglyceride remained even after adjustment for PE [13]. Additionally, the women with PE in our cohort all had late-onset PE whereas in the previous study, the participants likely suffered from early-onset PE since they delivered at a mean of 30 weeks gestation. In obesity, circulating FGF21 is increased [34]. The large inter-individual variability in FGF21 levels may also contribute especially with the relatively limited sample sizes of the studies to date.

FGF21 has been independently positively associated with hypertension in a community sample [29]. In our study, circulating maternal FGF21 was positively correlated with systolic blood pressure at enrolment and there was a trend for a correlation with diastolic blood pressure. This is a cross-sectional study and no causality can be inferred from our results. However, FGF21 levels have previously been associated with activity of the renin-angiotensin system in patients with end-stage renal disease on dialysis [35]. FGF21 was negatively correlated with residual renal function. Treatment with angiotensin receptor blockers reduced serum FGF21 concentrations independent of the inflammatory and insulin resistant-state. The FGF21 levels in this study were positively correlated to the level of insulin resistance in these dialysis patients independent of residual renal function, indicating that the regulation of FGF21 is complex.

A recent study showed the presence of low levels of FGF21 in the cord blood of 93% of their cohort of term and preterm infants [36]. The reported levels were at the threshold for detection for our assay and there was again a wide interindividual spread in the levels of FGF21. There was no difference in serum FGF21 in cord blood between term and preterm infants suggesting that FGF21 does not affect prenatal growth. This study reported a negative correlation between FGF21 levels and growth in length but not weight over the first six months of life [36], indicating that postnatally FGF21 may be a negative regulator of growth.

## Conclusions

In summary, late-onset preeclampsia is not associated with major changes to the expression of FGF21 or its receptors. The associations of FGF21 with the transcriptional regulator PPARα observed in placentas from women with gestational diabetes mellitus was not observed in placentas from women with late-onset preeclampsia. Gene expression levels of glucose transporters, which are targets of FGF21 signaling, were not affected by preeclampsia.

## Additional files

Additional file 1: Table S1. Primer sequences. Additional file 2: Figure S1. FGF21 localization in decidual cells.

## Abbreviations

FGF21: Fibroblast growth factor 21
GLUT: Glucose transporter
PE: Preeclampsia
