# Supplementary material for: Placental fibroblast growth factor 21 is not altered in late-onset preeclampsia
Source: Reprod Biol Endocrinol. 2015 Mar 8;13:14. doi: 10.1186/s12958-015-0006-3 (PMC4384232; doi:10.1186/s12958-015-0006-3)
Supplement: Additional file 1: Table S1. — Primer sequences. [file 12958_2015_6_MOESM1_ESM.docx]

Table S1 Primer sequences

| Gene name | Forward primer | Reverse primer | Amplicon length |
| --- | --- | --- | --- |
| *FGF21* | TGGATCGCTCCACTTTGACC | GGGCTTCGGACTGGTAAACA | 86 |
| *FGFR1* | GTCCAGCCCCTGCTATCTCA | CCTTTTCAAACTGACCCTTGCC | 146 |
| *FGFR2* | CACATTAGACCCAGAAGATGCC | GGCAGCGAAACTTGACAGTG | 179 |
| *FGFR3* | CCGTGCTCAAGTCCTGGAT | GATGCCTGCATACACACTGC | 215 |
| *FGFR4* | TCTGGAGTCCCGGAAGTGTA | TAGTCAATGTGGTGGACGCC | 120 |
| *KLB* | GGCTTTGAATGGCAGGATGC | CCACAGACTCGGGCTTAAGAA | 161 |
| *PPARA* | GTGGACTCAACAGTTTGTGGC | CCGAGCTCCAAGCTACTCTT | 155 |
| *PPARG* | GCAGTGGGGATGTCTCATAATGC | CAGGGGGGTGATGTGTTTGAA | 297 |
| *SLC2A1* | CACTGTCGTGTCGCTGTTTG | GGACCCTGGCTGAAGAGTTC | 237 |
| *SLC2A3* | TCCCAGCGAGACCCAGAGATGCT | TGGGGTGACCTTCTGTGTCCCCAT | 191 |
| *SLC2A4* | GAGGGGCCTGCCAGAAAGAGTCTG | CAGCTGCAGCACGACCGCAA | 180 |
| *CK7* | CCGTGCGCTCTGCCTATGGGG | GCTCCAGAAACCGCACCTTGTCGAT | 196 |
| *CD34* | CCACAGGAGAAAGGCTGGGCGA | AGCCCCTCGGTTCACACTGGC | 122 |
| *DES* | TCCGAGAAACCAGCCCTGAGCAA | GTGGCCTCACTGACGACCTCCC | 106 |
| *TBP* | GGGCACCACTCCACTGTAC | CTGTTCTTCACTCTTGGCTCCT | 289 |

*SLC2A1* encodes for GLUT1, *SLC2A3* encodes for GLUT3 and *SLC2A4* encodes for GLUT4.
