# Supplementary figures and images for: Placental fibroblast growth factor 21 is not altered in late-onset preeclampsia
Source: Reprod Biol Endocrinol. 2015 Mar 8;13:14. doi: 10.1186/s12958-015-0006-3 (PMC4384232; doi:10.1186/s12958-015-0006-3)

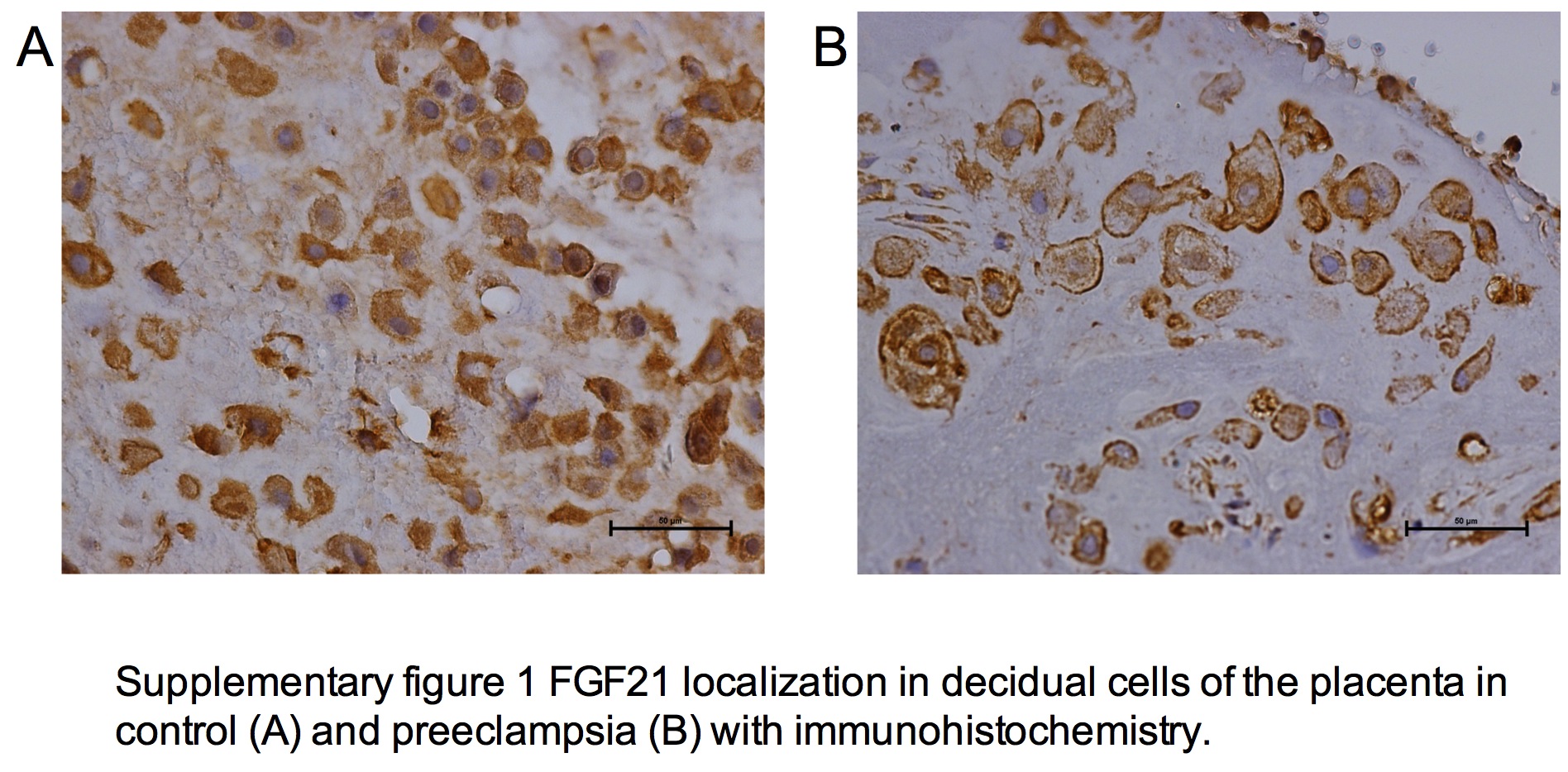

Supplement: Additional file 2: Figure S1. — FGF21 localization in decidual cells. [file 12958_2015_6_MOESM2_ESM.jpeg]
